# Dysfunction of estrogen-related receptor alpha-dependent hepatic VLDL secretion contributes to sex disparity in NAFLD/NASH development

**DOI:** 10.7150/thno.47037

**Published:** 2020-08-29

**Authors:** Meng Yang, Qingli Liu, Tongling Huang, Wenjuan Tan, Linbing Qu, Tianke Chen, Haobo Pan, Ling Chen, Jinsong Liu, Chi-Wai Wong, William W. Lu, Min Guan

**Affiliations:** 1 Center for Human Tissues and Organs Degeneration, Institute of Biomedicine and Biotechnology, Shenzhen Institutes of Advanced Technology, Chinese Academy of Sciences, Shenzhen 518055, Guangdong, China.; 2 University of Chinese Academy of Sciences, Beijing 100049, China.; 3 School of Life Sciences, Faculty of Science, The Chinese University of Hong Kong, Hong Kong, China.; 4 Guangzhou Institutes of Biomedicine and Health, Chinese Academy of Sciences, Guangzhou 510530, Guangdong, China.; 5 NeuMed Pharmaceuticals Limited, Yuen Long, Hong Kong, China.; 6 Department of Orthopaedics and Traumatology, The University of Hong Kong, Hong Kong, China.

**Keywords:** non-alcoholic fatty liver, non-alcoholic steatohepatitis, very low-density lipoprotein, estrogen-related receptor alpha, sex disparity

## Abstract

**Rationale:** Men and postmenopausal women are more prone to developing non-alcoholic fatty liver disease/steatohepatitis (NAFLD/NASH) than premenopausal women. However, the pathological links and underlying mechanisms of this disparity are still elusive. The sex-difference in hepatic very low-density lipoprotein (VLDL) assembly and secretion may contribute to NAFLD development. Estrogen-related receptor alpha (ERRα) is a key regulator of several metabolic processes. We hypothesized that ERRα plays a role contributing to the sex-difference in hepatic VLDL assembly and secretion.

**Methods:** VLDL secretion and essential genes governing said process were assessed in male and female mice. Liver-specific ERRα-deficient (ERRαLKO) mice were generated to assess the rate of hepatic VLDL secretion and alteration in target gene expression. Overexpression of either microsomal triglyceride transfer protein (*Mttp*) or phospholipase A2 G12B (*Pla2g12b*) by adenovirus was performed to test if the fatty liver phenotype in male ERRαLKO mice was due to defects in hepatic VLDL secretion. Female ERRαLKO mice were put on a diet high in saturated fat, fructose and cholesterol (HFHC) to promote NASH development. Wild type female mice were either ovariectomized or treated with tamoxifen to induce a state of estrogen deficiency or disruption in estrogen signaling. Adenovirus was used to overexpress ERRα in these mice to test if ERRα was sufficient to rescue the suppressed VLDL secretion due to estrogen dysfunction. Finally, wild type male mice on a high-fat diet (HFD) were treated with an ERRα inverse agonist to assess if suppressing ERRα activity pharmacologically would lead to fatty liver development.

**Results:** ERRα is an indispensable mediator modulating hepatic triglyceride-rich very low-density lipoprotein (VLDL-TG) assembly and secretion through coordinately controlling target genes apolipoprotein B (*Apob*), *Mttp* and *Pla2g12b* in a sex-different manner. Hepatic VLDL-TG secretion is blunted in ERRαLKO mice, leading to hepatosteatosis which exacerbates endoplasmic reticulum stress and inflammation paving ways for NASH development. Importantly, ERRα acts downstream of estrogen/ERα signaling in contributing to the sex-difference in hepatic VLDL secretion effecting hepatic lipid homeostasis.

**Conclusions:** Our results highlight ERRα as a key mediator which contributes to the sex disparity in NAFLD development, suggesting that selectively restoring ERRα activity in the liver may be a novel strategy for treating NAFLD/NASH.

## Introduction

One billion people worldwide are believed to have some form of non-alcoholic fatty liver disease (NAFLD) 1-3. While simple fatty liver can be reversed by diet and exercise, non-alcoholic steatohepatitis (NASH), a more severe form of NAFLD marked by inflammation and fibrosis, requires medical management to prevent its further deterioration into cirrhosis and even hepatocellular carcinoma (HCC) 4. Intriguingly, there is sex difference in terms of NAFLD incidence as men are more prone to developing NAFLD than premenopausal women 5. Importantly, the extent of fibrosis in NASH depends on hormonal status; namely, compared to premenopausal women, the extent of fibrosis is more severe in men and postmenopausal women 6-8. Understanding the molecular mechanisms that promote the sex disparity in NAFLD/NASH development should help pave ways to developing better therapeutic strategies 9, 10.

NASH development is currently thought to involve multiple perturbations, such as insulin resistance, hormones secreted from metabolic tissues 11, nutritional factors, gut microbiota as well as genetic and epigenetic factors, contributing to metabolic, oxidative, and inflammatory stresses 4. Excessive *de novo* lipogenesis (DNL) in the liver can be a key initiating factor contributing to NAFLD development. Little liver fat is evident as long as hepatic triglyceride (TG)-rich VLDL (VLDL-TG) secretion is sufficiently robust to help transport excess lipids from the liver to adipose tissue for storage or other metabolic tissues for consumption 12, 13. On the other hand, as the rate of DNL increases and exceeds that of hepatic VLDL secretion and fatty acid oxidation, excess lipids begin to build up in the liver, starting the process of fatty liver development 14. Intriguingly, dysfunctional VLDL synthesis and release is behind the transition from simple steatohepatosis to steatohepatitis 15-18.

Epidemiological and animal model studies have shown that female sex hormone estrogen is protective against NAFLD/NASH 7, 19, 20. Animal models of estrogen deficiency recapitulate several aspects of NASH development; specifically, restoring estrogen in ovariectomized (OVX) mice fed a high-fat and high-cholesterol (HFHC) diet blunts the development of NASH 19. Besides a clear NAFLD susceptibility difference between premenopausal and postmenopausal women, women with breast cancer undergoing anti-hormone treatments such as tamoxifen (TMX) or toremifene are prone to developing NAFLD and even NASH in obese patients with metabolic syndrome 20-23. In fact, those breast cancer patients who develop NAFLD on anti-estrogen have better disease free survival and overall survival compared to those without NAFLD, suggesting that this type of drug-induced NAFLD is strongly correlated to target engagement by anti-estrogen on ERα 24.

Estrogen-related receptor alpha (ERRα) is a nuclear hormone receptor that governs the expression of target genes involved in numerous metabolic pathways including those guiding mitochondrial oxidative phosphorylation and lipid metabolism 25. Although Estrogen-related receptor alpha (*Esrra*) is a direct target gene of estrogen receptor alpha (ERα) in breast cancer cells, ERRα-mediated metabolic alterations in response to estrogen/ERα signaling in liver tissue are elusive 26, 27. Here, we identify ERRα acts as a key mediator modifying the differential susceptibility to NAFLD/NASH development downstream of estrogen/ERα signaling. We find that higher hepatic ERRα expression levels in female mice accounts for elevating VLDL-TG secretion through coordinately controlling target genes apolipoprotein B (*Apob*), microsomal triglyceride transfer protein (*Mttp*) and phospholipase A_2_ G12B (*Pla2g12b*). Whereas, liver-specific deletion of ERRα diminishes this sex disparity and even leads to more severe fatty liver in female mice. Furthermore, we demonstrate that ERRα is indispensable for estrogen/ERα signaling for efficient VLDL-TG secretion and prevent NAFLD/NASH development under pharmacological manipulations and genetic modifications. Our results unravel a key role of ERRα linking sex hormone and hepatic lipid homeostasis, clarifying the mechanism underlying sex disparity in NAFLD/NASH development.

## Results

### Higher hepatic ERRα expression in female mice contributes to the sex disparity in VLDL-TG assembly and secretion

**Figure 1:**
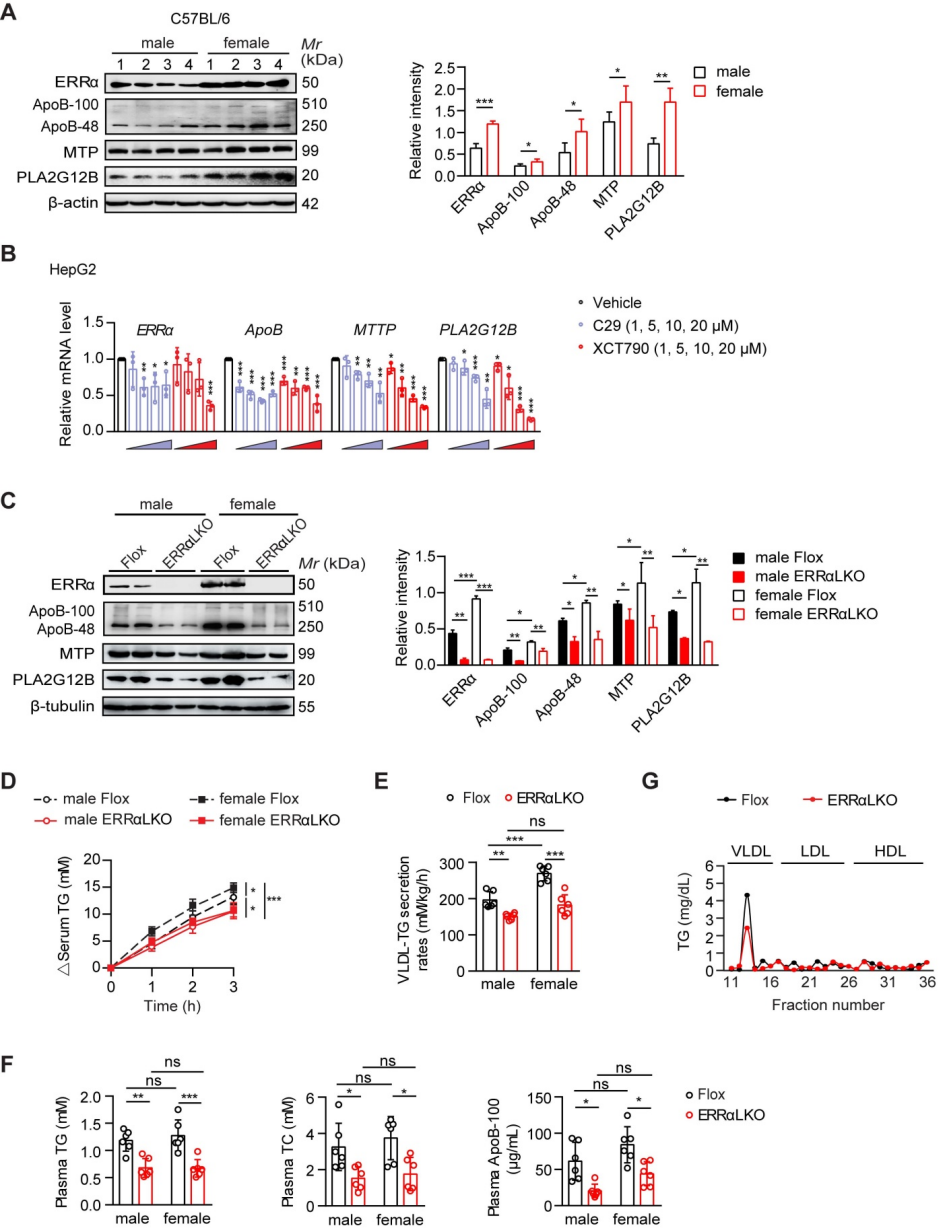
Higher hepatic ERRα expression in female mice contributes to the sex disparity in VLDL-TG secretion. (**A**) Western blot showing the hepatic expression of ERRα, ApoB, MTP and PLA2G12B in wild-type C57BL/6 mice at age of 12 weeks under 15 h fasting condition (left panel). Densitometry analysis of the western blotting data normalized to the intensity of β-actin (right panel). (**B**) mRNA levels of human* ESRRα*, *ApoB*, *MTTP* and *PLA2G12B* in HepG2 cells treated with different doses of ERRα inverse agonist C29 or XCT790 (*n* = 3). (**C**) Representative Western blot showing the hepatic protein expression of ERRα, ApoB, MTP and PLA2G12B in females and males of ERRα-flox (Flox) and liver-specific ERRα-deficient (ERRαLKO) mice at 9 weeks of age under 15 h fasting condition (left panel). Densitometry analysis of the western blotting data normalized to the intensity of β-tubulin (right panel). (**D-E**) Analysis of VLDL-TG secretion (D) and rates (E) in Flox and ERRαLKO mice fasted overnight followed by intravenous injection with 500 mg/kg body weight of Triton WR-1339 (*n* = 6 per condition and sex). (**F**) Levels of plasma TG, TC and ApoB-100 in mice as in (C). (**G**) Plasma lipoprotein profile analysis. VLDL, LDL and HDL fractions from 260 µL pooled plasma of Flox or ERRαLKO female and male mice fasted for 15 h were used for analysis. Data presented as means ± SD. *P< 0.05, **P < 0.01, ***P < 0.001, two-tailed Student's *t* test for paired groups (A-B) or one-way ANOVA followed by Tukey's *post hoc* analysis (C-F). Abbreviations: TG, triglyceride; TC, total cholesterol; VLDL, very low-density lipoprotein.

Premenopausal and lean women have both greater VLDL-TG secretion and clearance compared with lean men 12, 13. In the fasting state, most circulating plasma TG are in VLDL, which are synthesized and secreted by the liver. While there is no difference in plasma TG between overnight fasted male and female C57BL/6 mice, the sex difference in hepatic VLDL secretion rate between male and female C57BL/6 mice was recapitulated by injecting Triton WR-1339 to inhibit VLDL catabolism (Figure S1A-B). Notably, ERRα was found to be more highly expressed in the liver of female mice in the fasting state (Figure 1A). Several essential genes including* Apob* 28, *Mttp* 29, and *Pla2g12b* 30-32 involved in VLDL production and secretion were also found to be expressed in a sex-difference manner with *Pla2g12b* being the most obvious (Figure 1A). Two well-studied ERRα specific inverse agonists Compound 29 (C29) 33, 34 and XCT790 35 dose-dependently suppressed both the mRNA and protein expression levels of human ApoB, MTP and PLA2G12B in HepG2 cells (Figure 1B; Figure S1C). Besides, the expression of several other genes implicated in hepatic VLDL synthesis and assembly such as cell death-inducing DFFA-like effector b (*CIDEB*) 36, diacylglycerol O-acyltransferase 2 (*DGAT2*) 37, protein disulfide isomerase (*PDI*) 29, 38 were also found to be reduced (Figure S1D). These data imply that differential ERRα expression in the liver between female and male might account for the sex disparity in VLDL-TG secretion through fine tuning the expression of multiple essential genes associated with hepatic VLDL assembly and secretion.

In order to address whether ERRα plays a role in the higher rate of hepatic VLDL secretion in female, we generated liver-specific ERRα-deficient (ERRαLKO) mice and assessed their physiological and metabolic phenotypes (Figure S2A-H). ERRαLKO mice developed normally with no significant differences in body weight, food intake, body temperature, gonadal white adipose tissue (gWAT) and liver weight compared to control Flox littermates in both sexes (Figure S2C). In line with wild-type C57BL/6 mice, the expression levels of ERRα, ApoB, MTP, and PLA2G12B were found to be higher in female compared to male Flox mice, with ERRα and PLA2G12B being the most obvious (Figure 1C). In contrast, several essential genes including* Apob*, *Mttp* and *Pla2g12b* involved in VLDL production and secretion were quantified and found to be down-regulated in ERRαLKO mice (Figure 1C; Figure S2D-E). Consistently, hepatic VLDL-TG secretion was severely blunted in ERRαLKO mice (Figure 1D-E). Importantly, the sex disparity in VLDL secretion was diminished in ERRαLKO mice, highlighting an important role of ERRα contributing to said process (Figure 1D-E).

Plasma levels of TG and total cholesterol (TC) were decreased in ERRαLKO mice under fasted or fed condition (Figure 1F; Figure S2F). Plasma FFA and phospholipids levels were also lower in fasted ERRαLKO mice (Figure S2F). Importantly, the plasma level of ApoB-100 was reduced significantly in fasted ERRαLKO mice (Figure 1F; Figure S2G). Fractionation of plasma lipoproteins into VLDL, LDL and HDL by gel filtration revealed significantly lower levels of TG in the VLDL fractions of ERRαLKO mice (Figure 1G). There is no sex-difference of blood lipids levels in ERRαLKO mice (Figure 1F), suggesting that the circulating lipids levels might be further fine-tuned by uptake in other metabolic tissues and clearance by lipases, as previously shown in humans 12.

### Lack of ERRα in the liver accumulates more lipids in the female mice exacerbating NAFLD/NASH development

**Figure 2:**
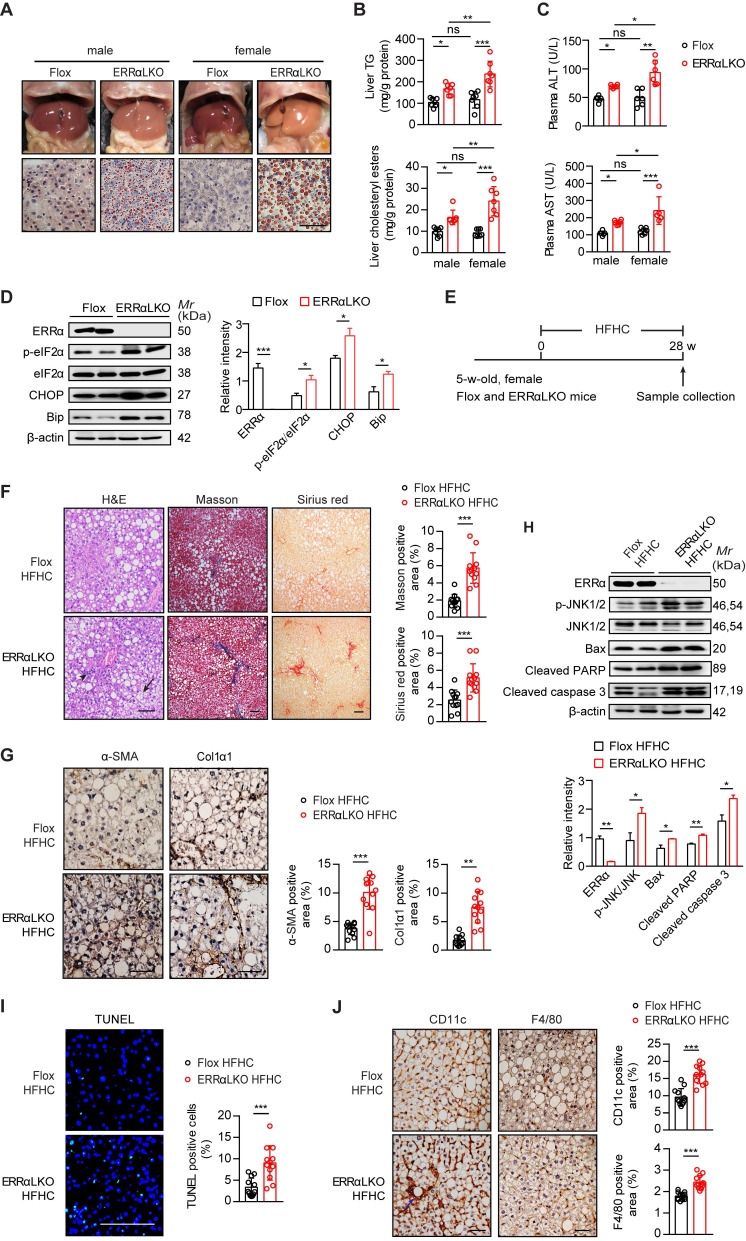
Effect of liver-specific ERRα deficiency on NAFLD/NASH development. (**A**) Representative images of liver tissue (top) and sections stained with Oil Red O (bottom) from Flox and ERRαLKO mice at 9 weeks of age. Scale bar, 50 µm. (**B**) Liver TG, cholesteryl esters levels in females and males of Flox and ERRαLKO mice under 15 h fasting condition (*n* = 7 mice per condition and sex). (**C**) Levels of plasma ALT and AST in mice as in (B), (n = 6 mice per condition and sex). (**D**) Western blots showing hepatic protein expression of endoplasmic reticulum stress markers phosphorylated eIF2α, CHOP and Bip from female Flox and ERRαLKO mice at 9 weeks of age on a normal chow diet (left panel). Densitometry analysis of the western blotting data normalized to the intensity of β-actin (right panel). (**E**) Schematic of the experimental procedure for NASH model (*n* = 6). (**F**) Representative images of H&E, Masson's trichrome, Sirius red-stained liver tissue sections from female Flox (top) and ERRαLKO (bottom) mice fed a HFHC for 28 weeks. Black arrow, ballooning degeneration of hepatocytes; arrowhead, infiltration of inflammatory cells. Percentages of the fibrotic area indicated by Masson's trichrome (blue) or Sirius red-positive (red) were calculated (*n* = 12 images). (**G**) Representative images showing immunohistochemical staining and quantification of α-SMA and Col1α1-positive cells (brown) in the liver sections (*n* = 12 images). (**H**) Western blots showing the hepatic protein levels of apoptosis associated markers phosphorylated JNK1/2, Bax, cleaved PARP and caspase 3 from female Flox and ERRαLKO mice fed a HFHC for 28 weeks (left panel). Densitometry analysis of the western blotting data normalized to the intensity of β-actin (right panel). (**I**) Representative images showing immunofluorescent staining and quantification of TUNEL-positive cells (green) in the liver sections (*n* = 12 images). (**J**) Representative images showing immunohistochemical staining and quantification of CD11c and F4/80-positive cells (brown) in the liver sections (*n* = 12 images). Blue arrow, crown-like structure. Scale bar, 100 µm. Data presented as means ± SD. *P< 0.05, **P < 0.01, ***P < 0.001, one-way ANOVA followed by Tukey's post hoc analysis (B-C) or two-tailed Student's *t* test (D, F-J). Abbreviations: TG, triglyceride; TC, total cholesterol; HFHC, high-fat and high-cholesterol diet; H&E, hematoxylin and eosin; Masson, Masson's trichrome.

Consistent with a blockage in VLDL production and secretion, ERRαLKO mice developed hepatosteatosis (Figure 2A). ERRαLKO primary hepatocytes accumulated more TG compared to floxed controls and the presence of oleic acid exacerbated such accumulation (Figure S3A-B). Intriguingly, Oil Red O staining of liver slides, liver TG and cholesterol showed male ERRαLKO mice developed mild fatty liver while the fatty liver phenotype was apparently more severe in female ERRαLKO mice (Figure 2A-B). The levels of plasma ALT and AST showed similar pattern consistent with the extent of fatty liver (Figure 2C). This sex disparity in liver fat content might be due to the higher reduction of hepatic VLDL secretion in female (32.8%) than male (23.6%) ERRαLKO mice (Figure 1D-E). HFD-induced hepatosteatosis is often more extensive in male than female mice 39, 40. A higher rate of hepatic VLDL-TG secretion in female maybe partly responsible for mobilizing more TG from the liver to be deposited in white adipose tissue; hence, protecting against the development of NAFLD at the expense of peripheral adiposity 12, 13. On the contrary, a context of female ERRαLKO mice with chronic compromised hepatic VLDL-TG secretion and more extensive lipids accumulation may easily lead to sustained cellular stress. As female ERRαLKO mice develop chronic hepatosteatosis even on a chow diet, endoplasmic reticulum stress markers such as chaperone GRP78/BiP and phosphorylated eukaryotic translation initiation factor 2α (eIF2α) were found to be elevated compared to Flox littermates (Figure 2D). Sustained endoplasmic reticulum stress in ERRαLKO also led to the induction of transcription factor C/EBP homologous protein (CHOP) (Figure 2D).

We next put female ERRαLKO mice on a diet high in saturated fat, fructose and cholesterol (HFHC) to examine if compromised hepatic VLDL-TG secretion accounts for exacerbated NASH development (Figure 2E). Compared to Flox littermates, female ERRαLKO mice on HFHC developed ballooning degeneration and extensive fibrosis revealed by H&E, Masson's trichrome and Sirius Red staining (Figure 2F). Hepatic stellate cells (HSC) trans-differentiation into activated myofibroblasts is believed to trigger hepatic fibrogenesis 41. HSC activation marker α-smooth muscle actin (α-SMA) and fibrosis marker collagen type I alpha 1 (Col1α1) were significantly induced in the liver of female ERRαLKO mice on HFHC by immunohistochemistry analysis (Figure 2G). Extensive liver cell death is also believed to play a critical role in NASH. Apoptosis associated markers such as phosphorylated JNK1/2, Bcl-2-associated X protein (Bax), cleaved Poly (ADP-ribose) polymerase (PARP), and caspase 3 were all elevated in the liver of female ERRαLKO mice on HFHC (Figure 2H). Consistent with these cellular markers of apoptosis, TUNEL staining revealed more extensive liver cell death (Figure 2I). The number of hepatic macrophages, characterized by F4/80 surface marker, were found to be elevated in the liver of female ERRαLKO mice on HFHC (Figure 2J). CD11c^+^ macrophages can form hepatic crown-like structure (hCLS) surrounding dead or dying hepatocytes with large lipid droplets. These hCLSs were also more readily detected in the liver of female ERRαLKO mice on HFHC (Figure 2J). Collectively, these data strongly suggest that loss of hepatic ERRα together with dietary insults can constitute physiological and nutritional perturbations triggering metabolic and cellular stresses, inflammation, fibrosis and even apoptosis that are relevant for NASH development.

### Rescued VLDL secretion in ERRαLKO mice are essential to preventing hepatosteatosis

**Figure 3:**
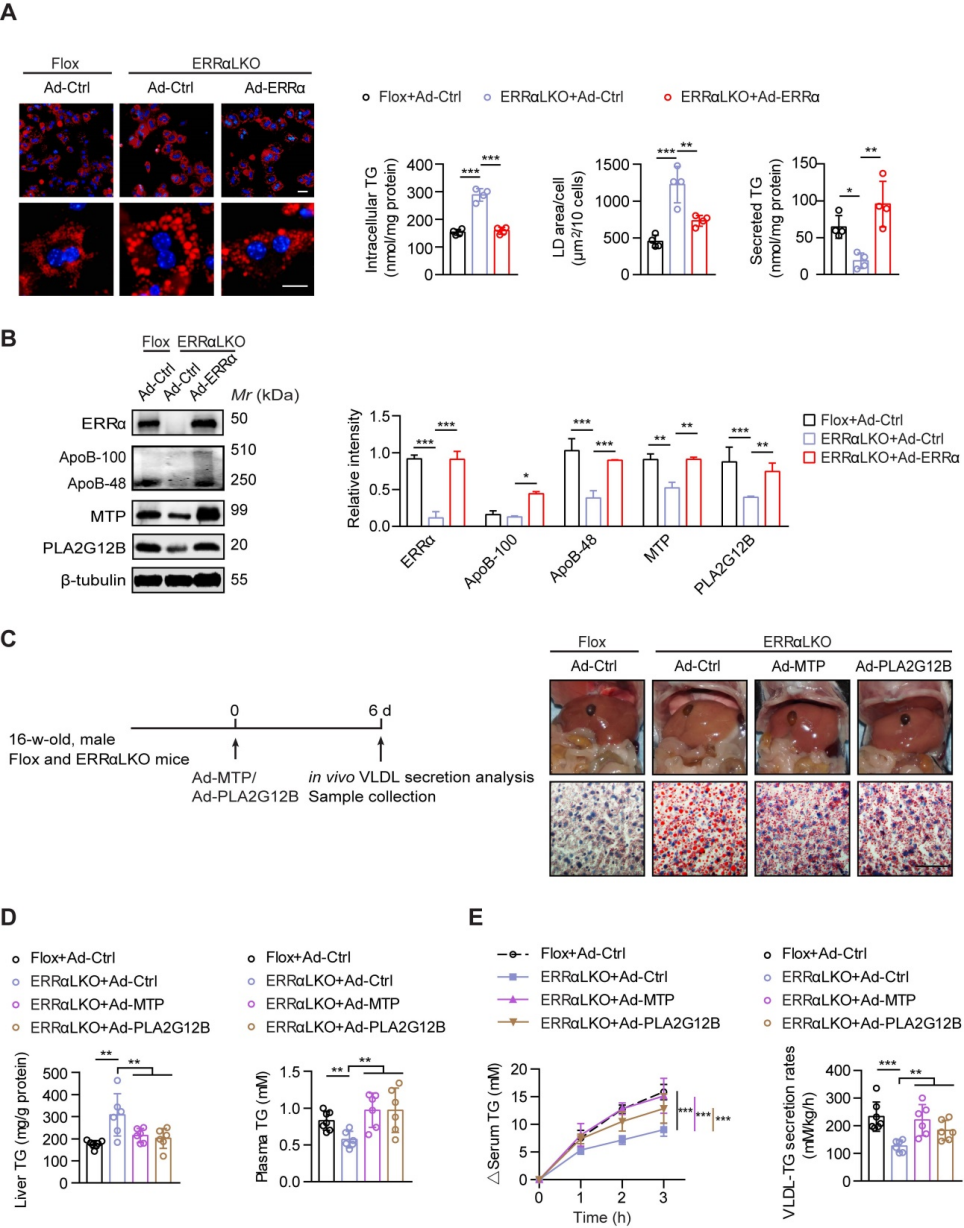
Rescued hepatic VLDL-TG secretion in ERRαLKO mice are essential to preventing hepatosteatosis. (**A**) Representative fields of male Flox and ERRαLKO hepatocytes infected with adenovirus expressing ERRα (Ad-ERRα) or control adenovirus (Ad-Ctrl) for 36 h. Lipid droplets (LDs) were labeled with Nile Red (red). Scale bar, 20 µm. Intracellular TG contents, average LD area and secreted TG from Flox and ERRαLKO hepatocytes infected with Ad-ERRα or Ad-Ctrl (*n* = 4). (**B**) Levels of proteins in male Flox and ERRαLKO hepatocytes infected with Ad-ERRα or Ad-Ctrl (left panel). Densitometry analysis of the western blotting data normalized to the intensity of β-tubulin (right panel). (**C and D**) Schematic of the experimental procedure of Ad-MTP, Ad-PLA2G12B or Ad-Ctrl-injected male Flox and ERRαLKO mice (*n* = 6-7 mice per group). Representative images of liver tissue (top), sections stained with Oil Red O (bottom) (C), liver TG and plasma TG levels (D) from indicated mice. Scale bar, 50 µm. (**E**) Analysis of VLDL-TG secretion (left panel) and rates (right panel) in treated mice (*n* = 6-7 mice per group). Data presented as means ±SD. *P< 0.05, **P < 0.01, ***P < 0.001, one-way ANOVA followed by Tukey's post hoc analysis (A-B) or two-tailed Student's *t* test (D-E).

Consistent with prior studies 42, 43, reduced expression of fatty acids oxidation genes and elevated expression of lipogenic genes were found in the liver of ERRαLKO mice which may contribute to lipids accumulation (Figure S3C). To further ascertain how ERRα governs hepatic VLDL secretion, we rescued ERRα expression by adenovirus in ERRαLKO primary hepatocytes. The amount of TG secreted into the medium was reduced in ERRαLKO primary hepatocytes compared to Flox counterparts (Figure 3A). In contrast, restoration of ERRα significantly alleviated TG accumulation and reduced lipid droplet size predominantly via restoring TG secretion into the medium (Figure 3A). In accordance with these observations, ectopic ERRα expression is capable of inducing the expression of ApoB, MTP, and PLA2G12B (Figure 3B). We next examine if rescuing the expression of these genes could remedy VLDL dysregulation caused by ERRα deficiency. Overexpression of MTP or PLA2G12B by adenovirus was sufficient to alleviate the lipids accumulation defect of ERRαLKO primary hepatocytes (Figure S4). Additionally, restoration of MTP or PLA2G12B expression in male ERRαLKO mice partially rescued the fatty liver phenotype and elevated hepatic VLDL-TG secretion (Figure 3C-E). MTP or PLA2G12B only affects VLDL packaging and secretion but not fatty acids oxidation or lipogenesis. Nonetheless, overexpression of either is capable of rescuing the fatty liver phenotype and restoring hepatic VLDL secretion in ERRαLKO mice (Figure 3C-E), strongly suggesting that dysfunction in hepatic VLDL secretion contributes to the disparity in the development of NAFLD in these ERRαLKO mice. Thus, these data suggest that ERRα is in charge of VLDL assembly and secretion acting upstream of the above genes.

### ERRα-mediated hepatic VLDL-TG secretion protects against NAFLD induced by estrogen deficiency in female mice

**Figure 4:**
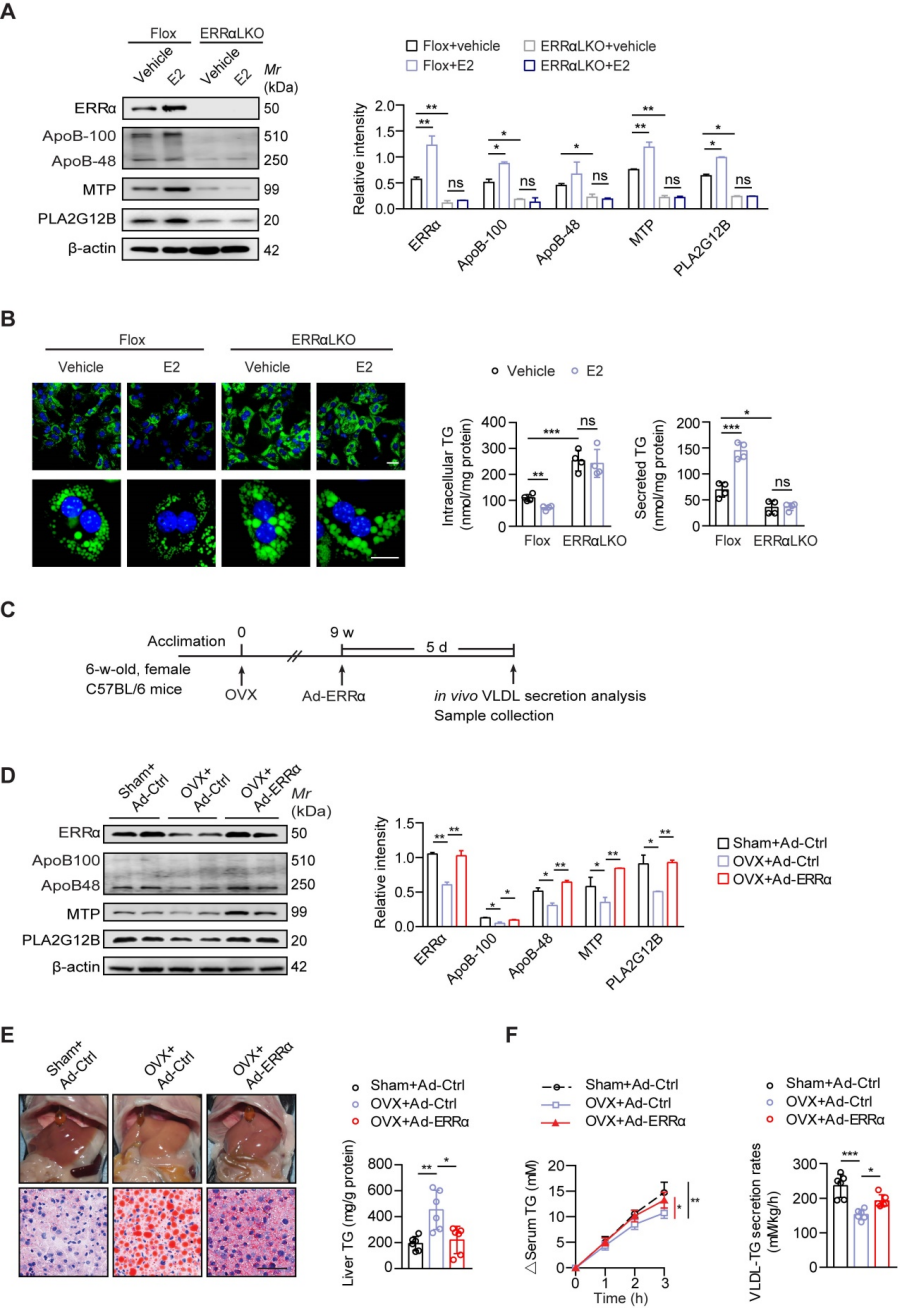
ERRα-mediated hepatic VLDL-TG secretion protects against NAFLD induced by estrogen deficiency. (**A**) Immunoblot analysis of ERRα, ApoB, MTP and PLA2G12B in female Flox and ERRαLKO hepatocytes with treatment of E2 (left panel). Densitometry analysis of the western blotting data normalized to the intensity of β-actin (right panel). (**B**) Representative fields, intracellular TG contents and secreted TG of female Flox and ERRαLKO hepatocytes treated with E2 (*n* = 4). LDs were labeled with BODIPY493/503 (green). Scale bar, 20 µm. (**C**) Schematic of the experimental procedure of OVX-induced NAFLD. (**D**) Western blots showing the indicated proteins expression in liver tissue from OVX or sham mice infected with Ad-ERRα or Ad-Ctrl (left panel). Densitometry analysis of the western blotting data normalized to the intensity of β-actin (right panel). (**E**) Representative images of liver tissue (top) and sections stained with Oil Red O (bottom) and liver TG of OVX or sham mice infected with Ad-ERRα or Ad-Ctrl (*n* = 6 mice). Scale bar, 50 µm. (**F**) Analysis of VLDL-TG secretion (left panel) and rates (right panel) in OVX or sham mice infected with Ad-ERRα or Ad-Ctrl (*n* = 6 mice). Data presented as means ± SD. *P < 0.05, ** P < 0.01, ***P < 0.001, one-way ANOVA followed by Tukey's *post hoc*. Abbreviations: OVX, ovariectomy; TG, triglyceride.

Postmenopausal women are more at risk of developing NAFLD/NASH than premenopausal women indicating a protective role of endogenous estrogen 6, 7. To examine if ERRα-mediated hepatic VLDL-TG secretion is required for the protective effects of estrogen, we first treated primary hepatocytes from female Flox and ERRαLKO with 17β-estradiol (E2). E2 treatment could enhance the expression of ERRα and VLDL-related genes such as *Apob*, *Mttp* and *Pla2g12b*, whereas the induction was blocked with loss of ERRα in primary hepatocytes (Figure 4A). Importantly, the ability of E2 to reduce TG accumulation and promote TG secreted into the media in primary hepatocytes was completely dependent on the presence of ERRα (Figure 4B).

Ovariectomized (OVX) female mice are widely used as an animal model to study risk factors associated with menopause; particularly, estrogen replacement has been shown to blunt this OVX-induced fatty liver 19, 44. To examine if higher levels of ERRα are essential for estrogen protection against NAFLD *in vivo*, OVX female wild-type mice were infected with either control or ERRα adenovirus (Figure 4C). Indeed, hepatic ERRα expression as well as ApoB, MTP, and PLA2G12B were found to be reduced in OVX mice compared to sham control (Figure 4D). Enforced ERRα expression by adenovirus reversed this OVX-blunted expression (Figure 4D). Furthermore, OVX mice developed fatty liver which was essentially reversed by ERRα overexpression (Figure 4E). Consistently, the OVX-suppressed hepatic TG secretion was also rescued by ERRα overexpression (Figure 4F), indicating that ERRα is an essential part of the estrogen protective mechanism against fatty liver development.

### ERRα is an indispensable mediator downstream of estrogen/ERα signaling in transcriptional regulation of VLDL-related genes

**Figure 5:**
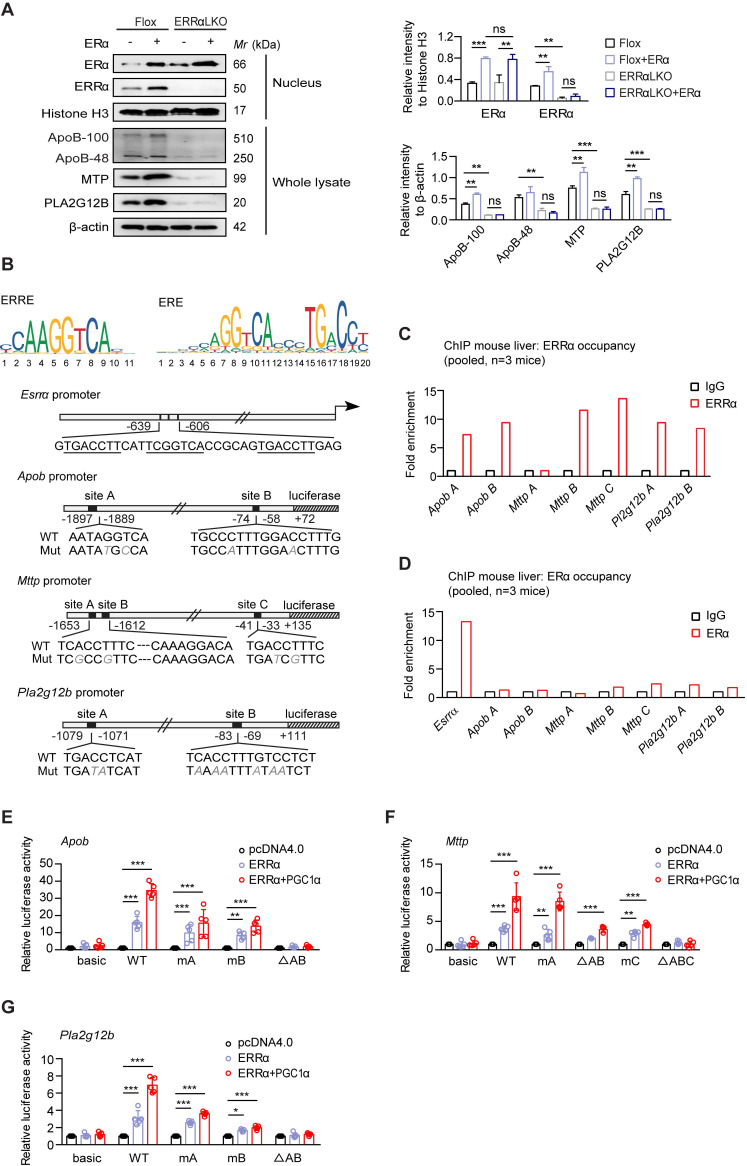
ERRα acts as an indispensable mediator of estrogen/ERα signaling in transcriptional regulation of VLDL-related genes. (**A**) Immunoblot analysis of ERα, ERRα, ApoB, MTP and PLA2G12B expression in female Flox and ERRαLKO hepatocytes in the presence of ERα transfection (left panel). Densitometry analysis of the western blotting data normalized to the intensity of Histone H3 or β-actin (right panel). (**B**) Top, sequence logos for ERRE (left) and ERE (right). Bottom, schematic representations of wild-type mouse *Esrra*, *Apob*, *Mttp* and *Pla2g12b* promoters and mutation constructs. (**C-D**) Chromatin immunoprecipitation analysis to examine occupancy of ERRα (C) and ERα (D) at the indicated genes in liver tissue from female wild-type mice fasted overnight were determined by qRT-PCR. (**E-G**) Luciferase reporter assays showing effect of ERRα w/o its co-activator PGC-1α on promoter serial constructs in HEK293T cells. Each ratio was normalized to the control (pcDNA4.0 vector) (*n* = 5). Data presented as means ± SD. *P < 0.05, ** P < 0.01, ***P < 0.001, two-way ANOVA followed by Bonferroni *post* analysis. Abbreviations: ERRE, ERRα response elements; ERE, estrogen receptor response elements; Mut, mutation.

ERα is a nuclear hormone receptor that governs the expression of target genes in response binding of estrogen to its ligand binding domain 26, 27, 45. ERRα has shown to be a downstream target of ERα response to estrogen signaling in breast cancer and endometrial carcinoma cells 26, 46, 47. Next, we asked if the effect of ERRα-mediated hepatic TG secretion is regulated by ERα signaling in the liver. We found that ectopic expression of ERα induced the expression of ERRα and VLDL-related genes such as *Mttp*, *Pla2g12b* and *Apob*, whereas the induction effect was completely abrogated with loss of ERRα expression in primary hepatocytes (Figure 5A). These data suggest that ERRα is an indispensable mediator of estrogen/ERα signaling in regulating hepatic VLDL secretion.

We evaluated that ERα or ERRα as a transcription factor may directly regulate the expression of *Apob*, *Mttp* and *Pla2g12b* by binding to potential estrogen receptor response elements (EREs) or ERRα response elements (ERREs) on their corresponding promoters. A previous study has reported several ERE half-sites on *Esrra* promoter located at -639 to -606 bp 46 (Figure 5B). In addition, bioinformatics analysis suggested the presence of two to three putative EREs/ERREs on each of the *Apob*-, *Mttp*-, and *Pla2g12b*-promoter (Figure 5B). Chromatin immunoprecipitation (ChIP) analysis from normal female mouse liver revealed binding of endogenous ERRα to some of these ERREs (Figure 5C). In contrast, ChIP analysis confirmed that ERα while fully capable of binding to the ERE on the promoter of *Esrra*, did not bind to these EREs half-sites on *Apob*-, *Mttp*-, and *Pla2g12b*-promoter (Figure 5D). Furthermore, deletion and mutation analysis of these putative ERREs confirmed that the ERRα-driven expression of *Apob* and *Pla2g12b* depends on both ERREs (Figure 5E,G); whereas, the ERRE located at -1620 to -1612 bp and -41 to -33 bp were primarily responsible for the ERRα-driven expression of *Mttp* (Figure 5F). Collectively, the aforementioned data strongly indicate a novel physiological role of ERRα; namely, ERRα acts as an indispensable master regulator mediating hepatic VLDL-TG secretion through coordinately controlling target genes involved in VLDL assembly and secretion.

### A reduced expression of ERRα is behind the tamoxifen-induced NAFLD in female mice

**Figure 6:**
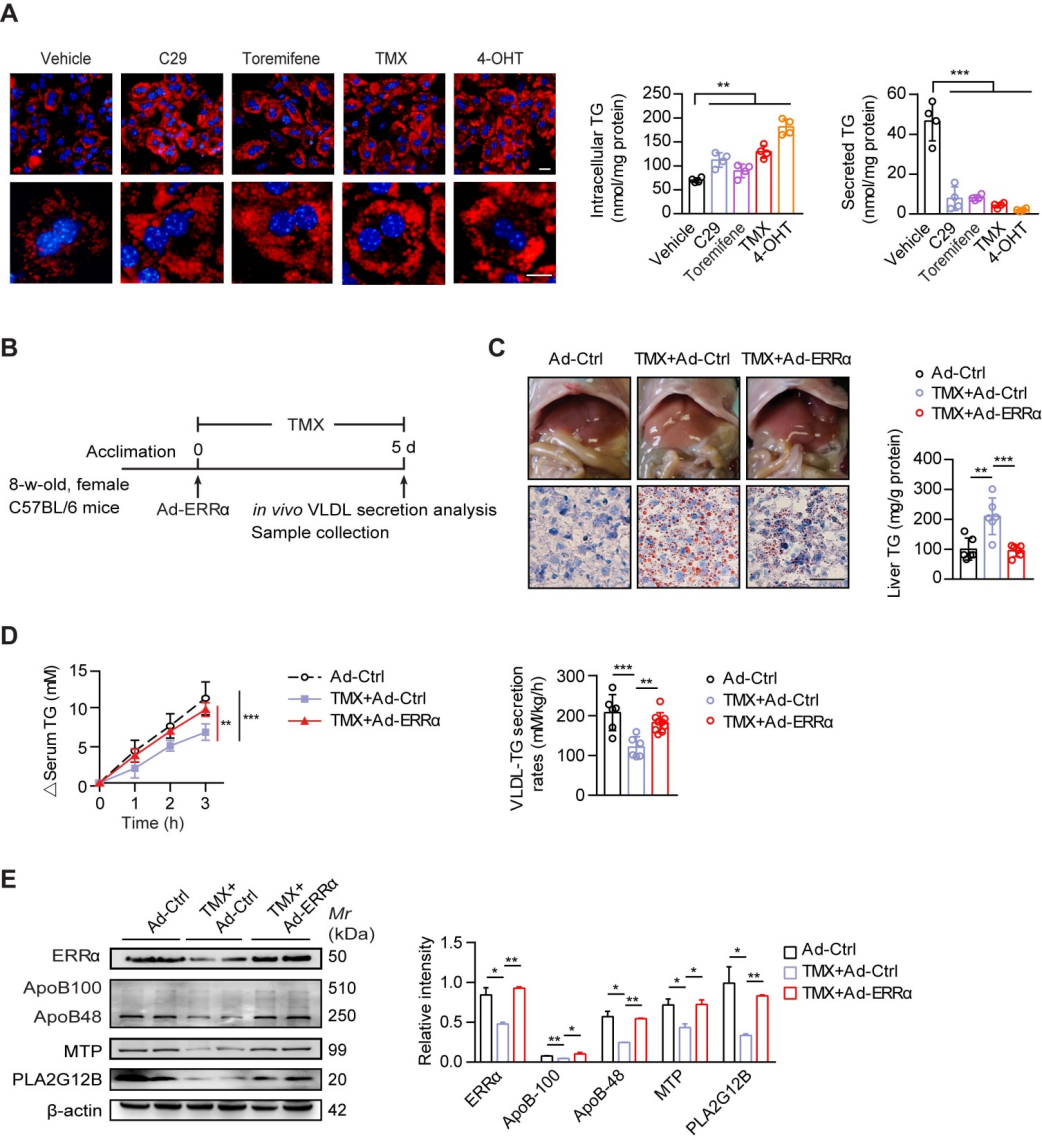
ERRα overexpression protects against TMX-induced hepatic VLDL dysfunction and NAFLD in female mice. (**A**) Representative fields, intracellular TG contents and secreted TG of female C57BL/6 mice hepatocytes treated with C29, TMX, toremifene and 4-OHT (*n* = 4). LDs were labeled with Nile Red (red). Scale bar, 20 µm. (**B**) Schematic of the experimental procedure of TMX-induced NAFLD in female mice. (**C**) Representative images of liver tissue (top), sections stained with Oil Red O (bottom) and liver TG levels from Ad-ERRα or Ad-Ctrl-injected female mice treated with TMX for 5 days (*n* = 6 mice). Scale bar, 50 µm. (**D**) Analysis of VLDL-TG secretion (left panel) and rates (right panel) in treated female mice (*n* = 6 mice per group for Ad-Ctrl and Ad-Ctrl+TMX; *n* = 9 mice for Ad-ERRα+TMX group). (**E**) Western blots showing the indicated protein expression in liver tissues from Ad-ERRα or Ad-Ctrl-injected female mice treated with TMX (left panel). Densitometry analysis of the western blotting data normalized to the intensity of β-actin (right panel). Data presented as means ± SD. **P < 0.01, ***P < 0.001, two-tailed Student's *t* test for paired groups (A), one-way ANOVA followed by Tukey's *post hoc* (C and E) or one-way ANOVA followed by Bonferroni *post hoc* analysis (D). Abbreviations: C29, compound 29; TMX, tamoxifen; 4-OHT, 4-hydroxytamoxifen; TG, triglyceride.

Tamoxifen (TMX) is used worldwide as a selective estrogen receptor modifier (SERM) for women with estrogen receptor-positive breast cancer by which treatment induces high risk of massive hepatic steatosis and even NASH 22, 23. Increased lipogenesis has been suggested as a primary mechanism. On the other hand, reduced hepatic TG-rich VLDL secretion has not been fully explored as another contributing factor 48. In light of the aforementioned evidence, we proposed that ERRα as a direct target of ERα may contribute to the TMX-induced NAFLD via additionally blunting hepatic VLDL assembly and secretion. Similar to ERRα inverse agonist C29, TMX and its hydrolyzed metabolite 4-hydroxytamoxifen (4-OHT) both led to TG accumulation in primary hepatocytes with reduced TG secretion (Figure 6A). Toremifene, another anti-estrogen that clinically has been shown to induce NAFLD in breast cancer patients, also modestly induced TG accumulation (Figure 6A). To examine if ectopic expression of ERRα would rescue the TMX-induced NAFLD *in vivo*, female mice treated with TMX were infected with either control or ERRα adenovirus (Figure 6B). Short-term TMX treatment induced mild fatty liver in mice infected with control adenovirus; whereas, over-expression of ERRα using adenovirus blocked such development (Figure 6C). Importantly, the TMX-suppressed hepatic VLDL-TG secretion as well as hepatic ApoB, MTP, and PLA2G12B expression were rescued by enforced ERRα expression (Figure 6D-E), strongly implying that reduced ERRα expression is a contributing factor behind the TMX-induced NAFLD.

### Suppressing ERRα activity exacerbates the development of NAFLD in male mice on a high-fat diet

**Figure 7:**
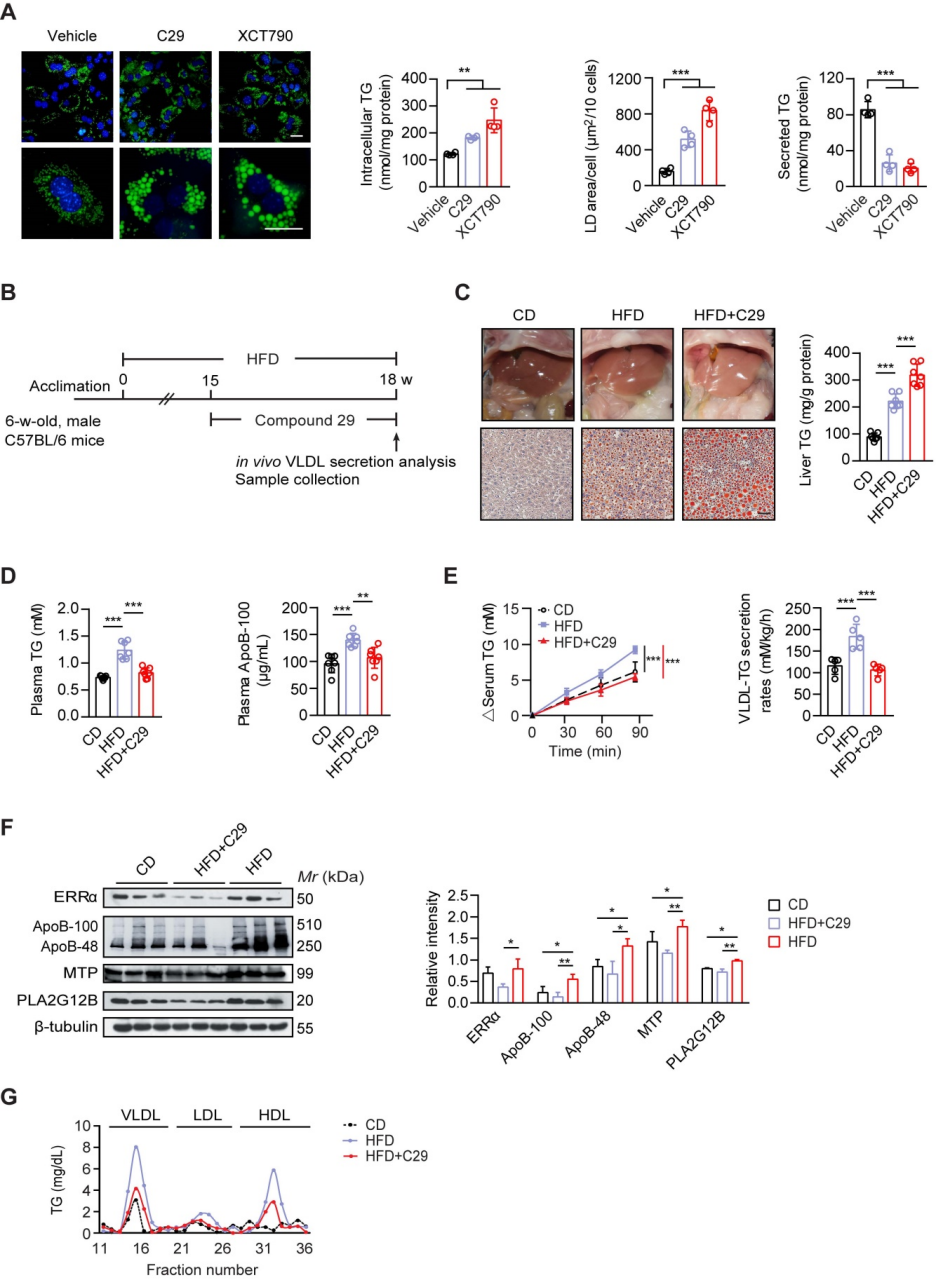
Suppressing ERRα activity exacerbates the development of NAFLD in male mice on a high-fat diet. (**A**) Representative fields of male C57BL/6 mice hepatocytes treated with ERRα inverse agonist C29 or XCT790. LDs were labeled with BODIPY493/503 (green). Scale bar, 20 µm. Intracellular TG contents, average LD area and secreted TG from male C57BL/6 mice hepatocytes treated with C29 or XCT790 (*n* = 4). (**B**) Schematic of the experimental procedure for HFD-induced NAFLD model. (**C-D**) Representative images of liver tissue (top) and sections stained with Oil Red O (bottom), liver TG (C), plasma TG and ApoB levels (D) from male mice fed a HFD or CD for 15 weeks followed by treatment with ERRα inverse agonist C29 for 3 weeks (*n* = 7 mice per group). Scale bar, 50 µm. (**E**) Analysis of VLDL-TG secretion (left panel) and rates (right panel) in male mice fed a HFD or CD followed by treatment with C29 (*n* = 5 mice per group). (**F**) Western blots showing the indicated protein expression in liver tissues from male mice fed a HFD or CD followed by treatment with C29 (left panel). Densitometry analysis of the western blotting data normalized to the intensity of β-tubulin (right panel). (**G**) Plasma lipoprotein profile analysis. VLDL, LDL and HDL fractions from 260 µL pooled plasma from male mice fed a HFD or CD followed by treatment with C29. Data presented as means ± SD. **P < 0.01, ***P < 0.001, two-tailed Student's *t* test for paired groups (A) or one-way ANOVA followed by Tukey's *post hoc* analysis (C-F). Abbreviations: HFD, high-fat diet; CD, chow diet; C29, Compound 29; TG, triglyceride.

Men are at a higher risk of NAFLD development compared to premenopausal women, particularly in the context of obesity 5, 6, 49. Since male ERRαLKO mice also develop mild fatty liver, we further asked whether suppressing ERRα activity would worsen NAFLD development in obese male. First, we verified that reducing ERRα activity by C29 and XCT-790 induced TG accumulation with large size lipid droplets in primary hepatocytes from male wild-type mice (Figure 7A); whereas, the amount of TG secreted into the media was considerably reduced (Figure 7A). Then, the specific ERRα inverse agonist C29 was administered to high-fat diet (HFD) induced obese male C57BL/6 mice (Figure 7B). Through Oil Red O staining of liver slices and quantification of liver TG, C29 was indeed demonstrated to exacerbate the HFD-induced hepatosteatosis *in vivo* (Figure 7C). On the other hand, C29 reduced the HFD-elevated plasma TG and ApoB levels as well as hepatic VLDL-TG secretion rate (Figure 7D-E), correlating to the suppressed hepatic expression of ApoB, MTP, and PLA2G12B by C29 (Figure 7F). Additionally, the HFD-induced higher levels of TG in VLDL and HDL factions were both decreased with treatment of C29 (Figure 7G). Thus, suppressing ERRα activity in the context of HFD may hasten NAFLD development.

## Discussion

NAFLD, a common prelude to cirrhosis and HCC, is the most common chronic liver disease worldwide. Men are more likely to develop NAFLD and HCC than women. Sex hormones such as estrogen and testosterone have differential effects on liver functions 50, 51. How this sex difference is governed mechanistically has not been thoroughly investigated. There are substantial sex differences in lipid metabolism, including the secretion and clearance of hepatic VLDL-TG. Here, we confirmed a similar sex disparity occurs in mice that strongly depend on the function of ERRα as liver-specific ERRαKO mice show no sex differences in hepatic VLDL secretion. We observed that ERRα acting downstream of estrogen/ERα signaling, is an indispensable mediator modulating hepatic VLDL-TG secretion through coordinately controlling target genes *Apob*, *Mttp* and *Pla2g12b* in a sex-different manner. Moreover, we found sex bias in NAFLD upon hepatic deficiency of ERRα apparently more severe in females paving way for NASH development. Importantly, disturbance of ERRα expression in female mice contributes to NAFLD/NASH development in physiological conditions such as estrogen deficiency and pharmacological conditions such as tamoxifen treatment.

As a transcription factor that targets multiple genes, ERRα is best known for its regulatory roles in mitochondrial biogenesis and oxidative phosphorylation 42, 43; therefore, loss of ERRα function is expected to have an impact on fatty acid oxidation. Besides these well-established roles, there are hints that ERRα has an impact on lipid synthesis as genes involved in fatty acid synthesis such as fatty acid synthase (*Fasn*) and sterol regulatory element binding protein-1 (*Srebp1*) 52 are upregulated in the liver of ERRα whole-body knockout mice 43. In our ERRαLKO mice, we additionally demonstrated that loss of ERRα function suppresses hepatic lipids secretion. All three of these aspects are believed to contribute to NAFLD with the contribution of each of these aspects varying under different physiological conditions. However, global ERRα knockout mice did not display NAFLD compared to our ERRαLKO mice 43, 53, 54. This discrepancy is likely due to reduced nutrient absorption in global ERRα knockout mice. Importantly, the significantly decreased serum lipids of ERRαLKO mice implies that hepatic lipids secretion strongly correlates to NAFLD development in these animals.

While the action of estrogen is generally believed to be more relevant in reproductive tissues, ERα is nonetheless expressed in the liver and exerts an influence on multiple metabolic pathways 45. Consistent with it having a liver protective role, both whole body and liver-specific ERα-deficient mice develop hepatosteatosis upon a high-fat diet with increased lipogenesis being an important contributing factor 55. Factors such as loss of protective estrogen after menopause or reduced ERα activity upon anti-estrogen treatment contribute to down-regulating hepatic VLDL-TG secretion; thereby, becoming a major culprit that accelerates the development of NAFLD 48. This process once becomes chronic cascades into a downward spiral characterized by endoplasmic reticulum stress and inflammation which are believed to be major culprits of NASH. ERRα shares functional features with ERα and its activity is modulated by the ERBB2 signaling pathway. However, ERRα and ERα display strict binding site specificity and maintain independent mechanisms of transcriptional activation 56. In this study, we demonstrated that ERRα acting downstream of estrogen/ERα signaling modulates hepatic TG-rich VLDL secretion through coordinately controlling target genes involved in this pathway. Specifically, ERRα binds to ERREs in the promoters of *Apob*, *Mttp,* and *Pla2g12b* and controls the expression of these rate limiting genes; thereby, dictating hepatic VLDL-TG assembly and secretion. Others genes in this pathway such as *Cideb*, *Dgat2*, and *Pdi* also harbor putative ERREs in their promoters and are likely to be coordinately regulated by ERRα. ERRα plays a key role in lipid homeostasis in the liver through promoting its oxidative phosphorylation, suppressing its synthesis, and promoting its clearance via elevating VLDL secretion. ERRα is thus protecting against excessive lipid accumulation in the liver and preventing lipid toxicity from causing metabolic stresses and inflammatory damages. This study reveals that ERRα is another important contributor to the sex difference in hepatic metabolic profiles, especially indispensable for estrogen/ERα signaling in governing hepatic VLDL secretion.

Conditions that lead to suppressed hepatic ERRα expression or activity level are probable NAFLD risks. For instance, postmenopausal women are known to have a higher risk for NAFLD compared to premenopausal women 6. While the hepatic ERRα expression level between these groups of women has not been investigated, hepatic ERRα expression is reduced in OVX female mice coinciding with hepatosteatosis. Thus, restoring the reduced hepatic ERRα expression or activity level in postmenopausal women may confer protective effects against NAFLD. Anti-estrogen such as tamoxifen and tormifene can reduce ERRα expression through suppressing ERα; whereas, rapamycin has been demonstrated to suppress hepatic ERRα activity in a post-translation manner by destabilizing the ERRα protein 43. These three drugs are known clinically to increase NAFLD incidences 20, 22, 57. While these three drugs act indirectly to reduce ERRα expression level, we proved that *bona fide* inverse agonist C29 exacerbates hepatosteatosis in HFD mice in consistence with a previous study 33.

ERRα inverse agonists are being developed as potential novel treatments for cancers and diabetes 33. Based on the role of ERRα in directing hepatic VLDL-TG secretion, an important concern for these ERRα inverse agonists would be drug-induced NAFLD upon prolonged dosing; particularly, in females. On the other hand, restoring ERRα activity level may represent a novel approach to targeting NAFLD/NASH. Here, using mouse models, we demonstrated that adenovirus-mediated overexpression of ERRα rescued the TMX- and OVX-induced hepatosteatosis. These findings highlight a novel physiological role of ERRα contributing for the sex disparity in VLDL secretion, providing an insight that a therapeutic approach for fatty liver involved selectively restoring hepatic ERRα activity, especially suitable for postmenopausal women with early stage NAFLD.

## Conclusions

Our results demonstrated that ERRα is an indispensable mediator required for estrogen/ERα signaling modulating hepatic VLDL secretion through coordinately controlling target genes *Apob*, *Mttp* and *Pla2g12b*. The imbalance of ERRα-mediated VLDL secretion leading to hepatic lipid accumulation may render female more prone to developing NAFLD/NASH under certain pharmacological or pathophysiological conditions including SERM treatment and estrogen deficiency.

## Methods

### Cell culture

All cells were cultured at 37 °C in a humidified atmosphere containing 5% CO2. HEK-293T and HEK-293 cells were cultured in Dulbecco's Modified Eagle's Medium (DMEM, GIBCO, 11965092) supplemented with 10% fetal bovine serum (FBS). HepG2 cells (sex: male) were cultured in DMEM (GIBCO, 41500034) supplemented with 10% FBS. Primary hepatocytes were isolated from the livers of 10- to 12-week-old male or female mice using the collagenase perfusion method. Briefly, after the tissues were digested via a perfusion of collagenase type I solution (Sigma, V900892), the liver was excised, minced and filtered through a 70-μm cell strainer (BD Falcon, 352350). The resulting solution was centrifuged to collect hepatocytes. Primary hepatocytes were further separated and purified using percoll solutions (GE Healthcare, 17-0891-02). The hepatocytes were then seeded on collagen-coated plates in DMEM supplemented with 10% FBS and 1% P/S. For each hepatocyte preparation, cell viability was estimated by the exclusion of trypan blue.

### Animals

An ERRα knockout mouse was generated using a targeting vector that contained an upstream homology arm of the *Esrra* gene, a loxP sequence and an FNFL (Frt-Neo-Frt-Loxp) cassette flanking exon 2 of the *Esrra* gene and a downstream homology arm of the *Esrra* gene. This targeting vector, which also carried the shv-TK sequence, was electroporated into SCR012 ES cells, selected with G418 and GanC to reduce random integration and then screened for homologous recombinants by PCR and Southern blot analysis. Positive ES clones were injected into C57BL/6 blastocysts to achieve initial germ-line transmission. Prior to conducting experiments, the Neo cassette was removed by crossing with Flp deleter strain (B6.SJL-Tg (*ACTFLPe*) 9205Dym/J, Shanghai Model Organisms Center, Inc.). The mice were backcrossed to C57BL/6 and then crossed with Albumin-Cre mice (B6.Cg-Tg (*Alb-Cre*) 21Mgn/J, The Jackson Laboratory, strain 003574) to generate liver-specific ERRα-deficient mice (*Albumin-Cre-Esrra^flox/flox^*, referred as ERRαLKO), the littermates mice carrying the floxed *ERRα* allele (*Esrra^flox/flox^*) were referred as control (Flox) mice. Primer sequences for genotyping are listed in Table S1. Mice were housed in standard cages at 22 to 24 °C with a 12 h dark/light cycle and ad libitum access to water. All animal studies were approved by the Institutional Animal Care and Use Committee of Shenzhen Institutes of Advanced Technology, Chinese Academy of Sciences.

### Mouse NAFLD and NASH models

Tamoxifen induced NAFLD model: 8-week-old female C57BL/6 mice (Charles River Laboratories) received daily intragastric administration of tamoxifen citrate (100 mg/kg body weight) or vehicle controls (0.3% Tween 80, 3% propylene glycol, 18% PEG400, 39% water, 40% corn oil) for 5 days before sacrifice. OVX induced NAFLD model: 6-week-old female C57BL/6 mice were underwent bilateral ovariectomized for 9 weeks to develop hepatosteatosis. Control mice (Sham) were subjected to sham operation survival surgery. HFD induced NAFLD model: 6-week-old male C57BL/6 mice were maintained on high-fat diet (60% kcal from fat, Research Diets, D12492) for 15 weeks. Mice were randomly divided into two groups. Mice were dosed once a day with vehicle or C29 (30 mg/kg body weight) by intragastric administration for 3 weeks. The vehicle used in this study consisted of 10% vitamin E-TPGS, 20% PEG400 and 70% water. Additional animals were maintained on standard chow diet (CD) and during the course of the study were given vehicle orally once a day. For NASH diet feeding, 5-week-old female ERRαLKO or Flox mice were fed a diet containing 40% fat, 22% fructose, and 2% cholesterol (Research Diets, D09100301) for 28 weeks. Body weight was monitored every two weeks. Mice were fasted overnight and then samples were harvested for analysis.

### *In vivo* VLDL-TG secretion assay

Hepatic VLDL-TG secretion was determined by blocking VLDL catabolism with Triton WR-1339 as previously reported 30, 36. Mice were fasted overnight and then injected with Triton WR-1339 (500 mg/kg body weight, Sigma-Aldrich, T8761) through retro-orbital veins. Blood samples were drawn from the tail vein at appropriate time points after injection and used for TG measurements. Hepatic VLDL-TG secretion rate was calculated from the slope of the curve and expressed as mM/h per kg body weight.

### Plasma and liver lipids measurements

Mice were fasted overnight before being sacrificed. Blood was immediately centrifuged at 1,500 g at 4 °C for 15 min. Plasma TG, total cholesterol (TC) (Applygen), high-density lipoprotein cholesterol (HDL-cholesterol), low-density lipoprotein cholesterol (LDL-cholesterol), alanine aminotransferase (ALT) and aspartate aminotransferase (AST) were measured using commercial kits (Nanjing Jiancheng Bioengineering Institute). Plasma free fatty acid (FFA), phospholipids and apolipoprotein B were detected by kits (Mlbio). For lipoprotein analysis, 260 μL of plasma was fractioned by high performance liquid chromatography (HPLC) using a Superose 6 column (GE Healthcare, 17-5172-01). TG and cholesterol in each fraction were measured. Total hepatic lipids were extracted from the liver using the Folch method. Liver TG, cholesterol esters, FFA and phospholipids were measured using commercial kits as above according to the manufacturer's instructions.

### Adenovirus production and infection

To generate Ad-ERRα, Ad-MTP and Ad-PLA2G12B, ERRα, MTP or PLA2G12B cDNA was inserted into the pAdTrack-CMV vector and the pAd-Easy system (Stratagene) was used to build the virus. Adenoviruses were multiplied in HEK-293 cells and purified by CsCl density gradient ultracentrifugation. Adenoviruses were injected via the tail-veins at a dosage of 1×10^9^ plaque-forming units (pfu) /mouse. Mice were sacrificed 5-6 days post injection. For primary hepatocyte, cells were infected with adenoviruses at a multiplicity of infection of 100 pfu for 36 h, and then collected for further analysis.

### Lipid droplets and TG contents analysis in primary hepatocytes

Hepatocytes grown in medium supplemented with 100 μM oleic acid (Sigma, O1383) overnight to enhance the formation of large LDs. Cells were fixed with 3% formaldehyde for 15 min, and stained with BODIPY 493/503 (1μg/mL, Invitrogen, D-3922) or Nile Red (1μg/mL, Sigma, N3013) for 10 min at room temperature. Nuclei were stained using DAPI (Cell signaling, 4083) and mounted with prolong gold anti-fade reagent (Invitrogen, P10144) followed by washing in PBS for 3 times. The images were captured using Olympus fluorescence microscope. All images were generated using Image J software. Lipid droplet area was quantified in randomly chosen fields using the Particle analysis Plugin of the Image J software. The average lipid droplet area/10 cells was calculated by dividing the overall lipid droplet area by the number of 10 cells in the same field.

For intracellular TG content measurement, cells were replaced with fresh phenol-free media supplemented with 10% charcoal stripped FBS and incubated with either 17β-Estradiol (E2, 100 nM, MCE, HY-B0141), compounds 29 (C29, 20 μM), tamoxifen citrate (TMX, 20 μM, MCE, HY-13757), toremifene citrate (10 μM, TargetMol, T1464), 4-Hydroxytamoxifen (4-OHT, 10 μM, Sigma, H6278) or vehicle for 36 h. Intracellular TG were then extracted for detection. For TG secretion in hepatocytes, cells were treated for 30 h and replaced with medium without FBS, and then medium was collected after additional 5 h incubation. TG levels were assessed by TG quantification kit (Bioassay, ETGA-200). Protein concentrations in cell lysates were measured using a BCA Protein Assay Kit (Pierce, 23225). Intracellular and secreted TG content were expressed as nanomoles of lipid per microgram of total protein.

### Histological staining

Formalin-fixed, paraffin-embedded mouse liver sections were stained with hematoxylin and eosin (H&E), Masson's trichrome or Sirius red. Frozen liver slides were selected to perform Oil Red O staining. For immunohistochemistry, the liver sections were blocked in normal serum and incubated with α-SMA (Cell signaling, 19245S, lot# 1), Collagen I alpha 1 (Novus Biologicals, NBP1-30054, lot# SH317a), F4/80 (Abcam, ab6640, lot# GR3189625-1), and CD11c (Abcam, ab11029, lot# GR307454-12) at 4 °C overnight followed by incubating with Horseradish Peroxidase (HRP)-conjugated secondary antibodies and detecting with diaminobenzidine (DAB) and hematoxylin as the counter stain. The images were captured using an Olympus light microscope. Positive areas were measured using Image J software. Two random fields from each slide were selected for quantitation.

### TUNEL staining

The liver sections used for terminal deoxynucleotidyl transferase-mediated dUTP nick-end labeling (TUNEL) staining assays were prepared through fixation in 4% paraformaldehyde and soaked in 0.1% Triton X-100. The thickness of the liver sections was 5 µm. TUNEL staining was performed to evaluate liver injury using an *in situ* Cell Death Detection Kit (Roche, 11684795910) according to the manufacturer's instructions. The nuclei were labeled with DAPI. The images were captured using Olympus fluorescence microscope. TUNEL positive cells were quantified as the percentage of TUNEL positive cells per total DAPI-stained cells.

### RNA isolation and quantitative PCR analysis

Total RNA was isolated using trizol reagent (Invitrogen, 15596018) as described in the manufacturer's instructions. 2 μg of RNA was reverse transcribed using cDNA Transcription kit (Thermo, K1622) for first-strand cDNA synthesis utilizing an oligonucleotide dT primer and real time PCR was performed using SYBR Green (Toyobo, QPK-201). Housekeeping gene *Actin* was used to normalize in all the experiments. Primers used are listed in Table S2 and Table S3.

### Western blot analysis

Samples were lysed in radioimmune precipitation assay buffer supplemented with complete EDTA-free protease inhibitor (Roche, 4693159001) and 1 mM PMSF (Sigma, 78830). Protein fractions were quantified by a BCA protein assay kit according to the manufacturer's instructions. Samples were separated by SDS-PAGE and then transferred to PVDF membranes (Millipore, IPVH00010) blocked with nonfat milk. Membranes were blotted with different primary antibodies overnight at 4 °C. Antibodies for western blotting were anti-ERRα (Cell signaling, 13826, lot# 1, 1:1000), anti-ApoB (Abcam, 20737, lot# GR217652-5, 1:2500), anti-MTP (BD Transduction Laboratories, 612022, lot# 5205643, 1:1000), anti-PLA2G12B (Abbiotec, 252133, lot# 19081305, 1:2000), anti-ERα (Abcam, ab32063, lot# GR187736-37, 1:1000), anti-Histone H3 (Abcam, 1791, lot# GR3237728-1, 1:5000), anti-β-actin (Cell signaling, 3700, lot# 13, 1:1000), β-tubulin (BPI, AbM9005-37B-PU, lot# 201304, 1:15000), Albumin (Proteintech, 16475-1-AP, lot# 00018838, 1:5000), anti-peIF2α (Cell signaling, 3398S, lot# 6, 1:1000), anti-eIF2α (Cell signaling, 5324S, lot# 4, 1:1000), anti-Bip (Cell signaling, 3177S, lot# 9, 1:1000), anti-CHOP (Cell signaling, 2895S, lot# 11, 1:1000), anti-pJNK (Cell signaling, 4668, lot# 15, 1:1000), anti-JNK (Santa cruz, sc1648, 1:500), anti-Bax (Proteintech, 50599, lot# 00078538, 1:1000), anti-caspase 3 (Cell signaling, 9662S, lot# 19, 1:1000), anti-PARP (Cell signaling, 9532S, lot# 9, 1:1000). After incubation with corresponding secondary antibodies, blots were developed using an enhanced chemiluminescence kit (Millipore, WBLUR0500) and exposed in Amersham Imager 600 (GE Healthcare).

### Transient transfection and luciferase reporter assays

Human *PGC1α* and *ESRRα* were cloned into pcDNA4.0 vector. *Apob*, *Mttp,* and *Pla2g12b* promoter serial constructs were cloned and ligated into pGL3 control luciferase reporter vector. Mutations were introduced into putative ERRα binding sites by PCR-based site-directed mutagenesis using QuikChange Site-Directed Mutagenesis Kit (Stratagene, 200518). HEK-293T was maintained at 37 °C in DMEM containing 10% FBS, and 1% P/S. Serial constructs and site-directed mutagenesis primers are described in Table S4. For the luciferase reporter assays, medium was replaced with phenol free α-MEM containing 1% charcoal stripped FBS. All the transient transfections were conducted using lipofectamine 3000 (Invitrogen, L3000015) according to the manufacturer's instructions.

### Chromatin immunoprecipitation assay

Chromatin immunoprecipitation assay was using by Magna ChIP G Tissue Kit (Millipore, 17-20000). Livers from wild-type female mice fasted overnight were collected and mildly dissociated by dounce with pestle A for six strokes in PBS containing 1% formaldehyde and rocked for 15 min, quenched with glycine, washed with PBS, and sonicated with a probe-type sonifier in tissue lysis buffer supplemented with protease inhibitors and PMSF. Sonicated extracts contained 10 μg cross-linked chromatin were immunoprecipitated with antibodies for ERα (6.7 μL), ERRα (10 μL) or IgG (2 μL, Cell signaling, 2729, lot# 8), and captured with protein G magnetic beads. DNA was purified using spin columns. Three sets of input and immunoprecipitated samples from three mice were pooled. Sample was analyzed by real time PCR and the amount of immunoprecipitated DNA in each sample was presented as fold relative enrichment to IgG-associated DNA by using the delta Ct (ΔCt) method. Primer sequences are listed in Table S5.

### Statistical analyses

Data are expressed as mean ± SD. All data were analyzed using the appropriate statistical analysis methods with Microsoft Excel and/or GraphPad Prism. A two-tailed Student's *t* test was used for pairwise comparison of genotypes or treatments. One-way ANOVA or two-way ANOVA were used when comparing three or more groups, as indicated in the figure legends. *P* < 0.05 was considered to be significant, as indicated by asterisks in the figures.

## Supplementary Material

Supplementary figures and tables.

## Abbreviation

NAFLD: non-alcoholic fatty liver disease
VLDL: very low-density lipoprotein
NASH: non-alcoholic steatohepatitis
HCC: hepatocellular carcinoma
ERRα: estrogen-related receptor alpha
TG: triglyceride
ApoB: apolipoprotein B
MTP: microsomal triglyceride transfer protein
PLA2G12B: phospholipase A_2_ G12B
OVX: ovariectomized
TMX: tamoxifen
ERα: Estrogen receptor alpha
gWAT: gonadal white adipose tissue
FFA: free fatty acids
TC: total cholesterol
Cideb: cell death-inducing DFFA-like effector b
Dgat2: diacylglycerol O-acyltransferase 2
PDI: protein disulfide isomerase
C29: compound 29
E2: 17β-estradiol
ERREs: ERRα response elements
EREs: estrogen receptor response elements
4-OHT: 4-hydroxytamoxifen
HFD: high-fat diet
CHOP: C/EBP homologous protein
eIF2α: eukaryotic translation initiation factor 2α
HFHC: diet high in saturated fat, fructose and cholesterol
HSC: Hepatic stellate cells
α-SMA: α-smooth muscle actin
Col1α1: collagen type I alpha 1
Bax: Bcl-2-associated X protein
PARP: Poly (ADP-ribose) polymerase
hCLS: hepatic crown-like structure
Fasn: fatty acid synthase
Srebp1: sterol regulatory element binding protein-1
DNL: de *novo* lipogenesis
