# Supplementary material for: Dysfunction of estrogen-related receptor alpha-dependent hepatic VLDL secretion contributes to sex disparity in NAFLD/NASH development
Source: Theranostics. 2020 Aug 29;10(24):10874–91. doi: 10.7150/thno.47037 (PMC7532682; doi:10.7150/thno.47037)
Supplement: Supplementary file 1 — Supplementary figures and tables. [file thnov10p10874s1.pdf]

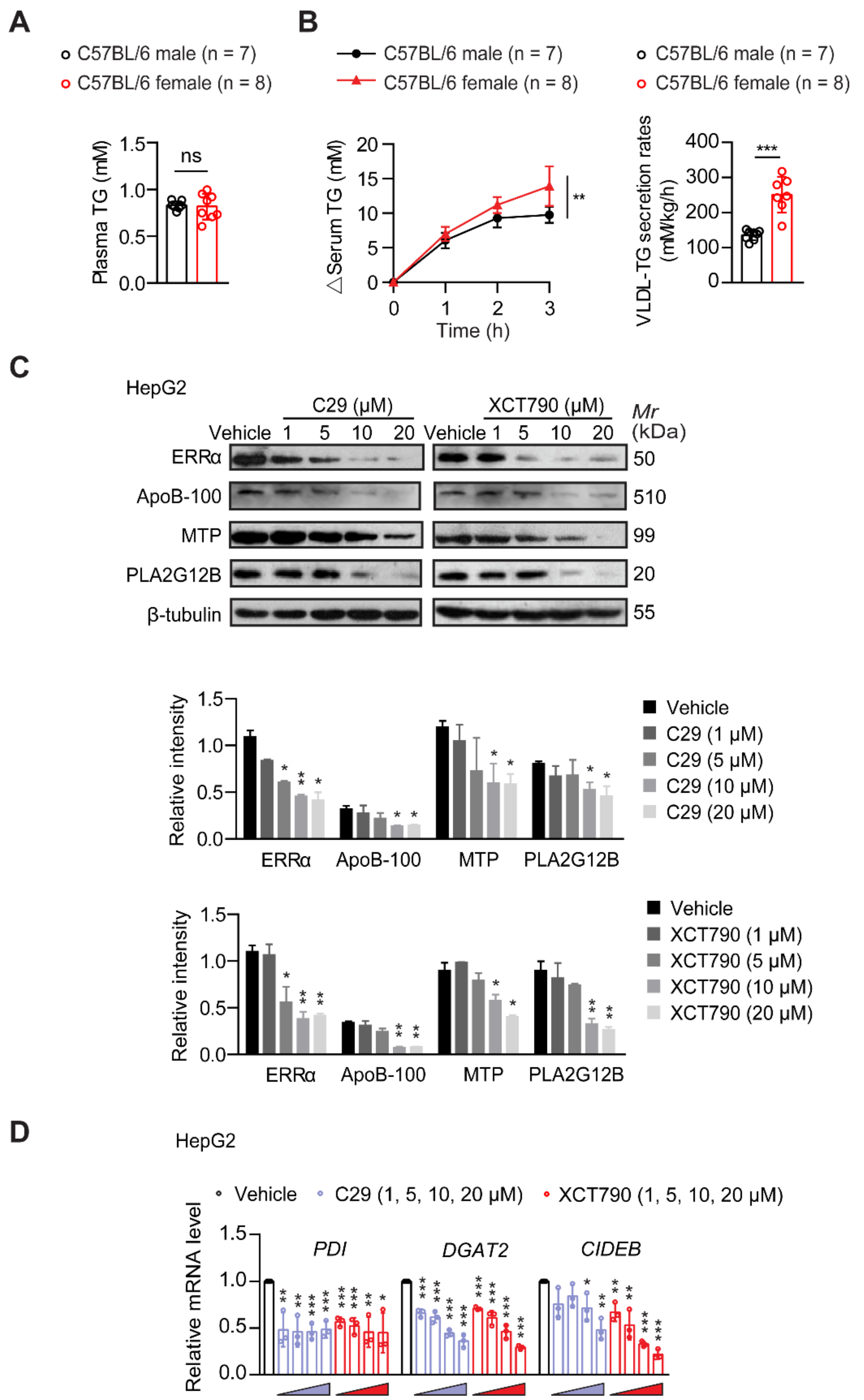

Figure S1. ERRα mediates gender disparity in hepatic VLDL secretion and associated genes. (A) Levels of plasma TG from 12-week-old male and female C57BL/6 mice fasted overnight. (B) VLDL-TG secretion (left panel) and rates (right panel) in 12-week-old C57BL/6 mice fasted overnight followed by intravenous injection with

500 mg/kg body weight of Triton WR-1339. (C) Protein levels of human ERRα, ApoB, MTP and PLA2G12B in HepG2 cells treated with different doses of ERRα inverse agonist C29 or XCT790. Densitometry analysis of the western blotting data normalized to the intensity of β-tubulin. (D) mRNA levels of human *PDI*, *DGAT2* and *CIDEB* in HepG2 cells treated as (C) (n = 3). Data presented as means ± SD. $^{*}P < 0.05$, $^{**}P < 0.01$, $^{***}P < 0.001$, two-tailed Student's t test.

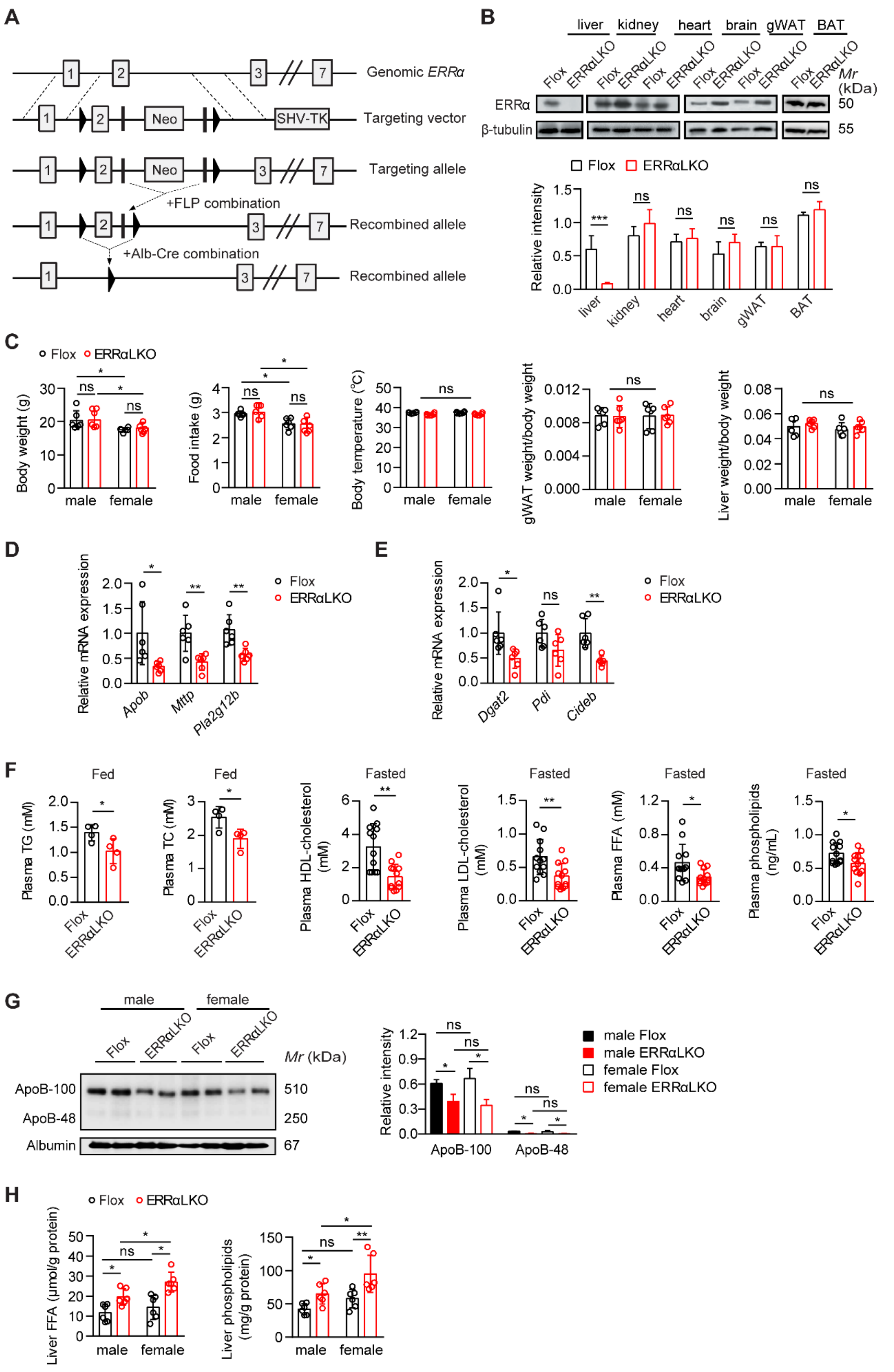

Figure S2. Generation and characterization of liver-specific ERRα-deficient mice. (A)

Schematic diagram of the strategy used to generate liver-specific ERRα-deficient mice. (B) Western blotting for ERRα in indicated tissues of control (Flox) and ERRαLKO mice (left panel). Densitometry analysis of the western blotting data normalized to the intensity of β-tubulin (right panel). (C) Body weight, food intake, body temperature, relative liver weight and gonadal white adipose tissue weight of male and female ERRαLKO mice compared with Flox littermates at 9 week of age (n = 6 mice per condition and sex). (D) mRNA levels of genes related to VLDL secretion (*Apob*, *Mttp* and *Pla2g12b*) in the livers of Flox and ERRαLKO mice (n = 6 mice per group). (E) mRNA levels of genes related to VLDL secretion (*Dgat2*, *Pdi* and *Cideb*) in the livers of Flox and ERRαLKO mice (n = 6 mice per group). (F) Levels of plasma TG, TC under fed condition and plasma HDL-cholesterol, LDL-cholesterol, FFA, phospholipids in Flox and ERRαLKO mice under fasting condition ($n = 4$ mice per group for fed condition and $n = 12$ mice per group for fasting condition). (G) Western blot showing plasma levels of ApoB protein from Flox and ERRαLKO male and female mice (left panel). Densitometry analysis of the western blotting data normalized to the intensity of Albumin (right panel). (H) FFA and phospholipids levels from liver of Flox and ERRαLKO mice under fasting condition (n = 6 mice per condition and sex). Data presented as means ± SD. $*P < 0.05$, $**P < 0.01$, two-tailed Student's t test for paired groups (B, D-F) or one-way ANOVA followed by Tukey's post hoc analysis (C, G and H). Abbreviations: gWAT, gonadal white adipose tissue; BAT, brown adipose tissue; FFA, free fatty acids; TG, triglyceride; TC, total cholesterol.

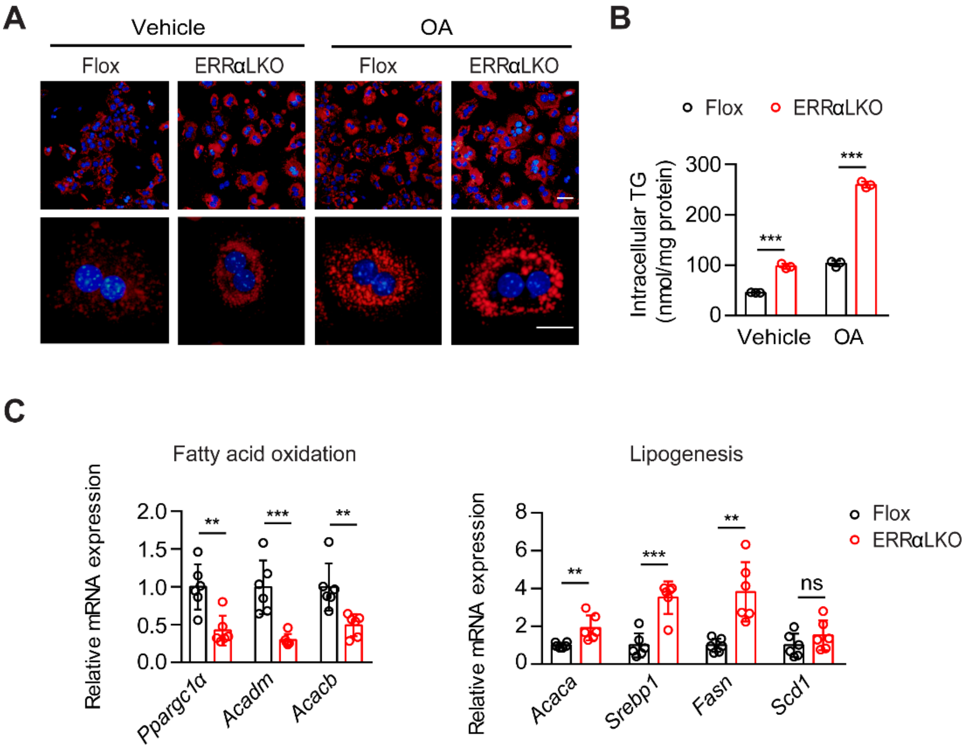

Figure S3. ERRα governs expression of essential genes involved in lipid metabolism. (A) Representative fields of primary hepatocytes from male Flox and ERRαLKO mice with or without OA treatment. Lipid droplets (LDs) were labeled with Nile Red (red). (B) Intracellular TG contents from Flox and ERRαLKO hepatocytes (n = 3). (C) mRNA levels of genes related to fatty acid oxidation (*Ppargc1α*, *Acadm* and *Acacb*) and genes related to lipogenesis (*Acaca*, *Srebp1*, *Fasn* and *Scd1*) in the livers of Flox and ERRαLKO mice (n = 6 mice per group). Data presented as means ± SD. $*P < 0.05$, $**P < 0.01$, $***P < 0.001$, two-tailed Student's t test. Abbreviations: OA, oleic acid; TG, triglyceride.

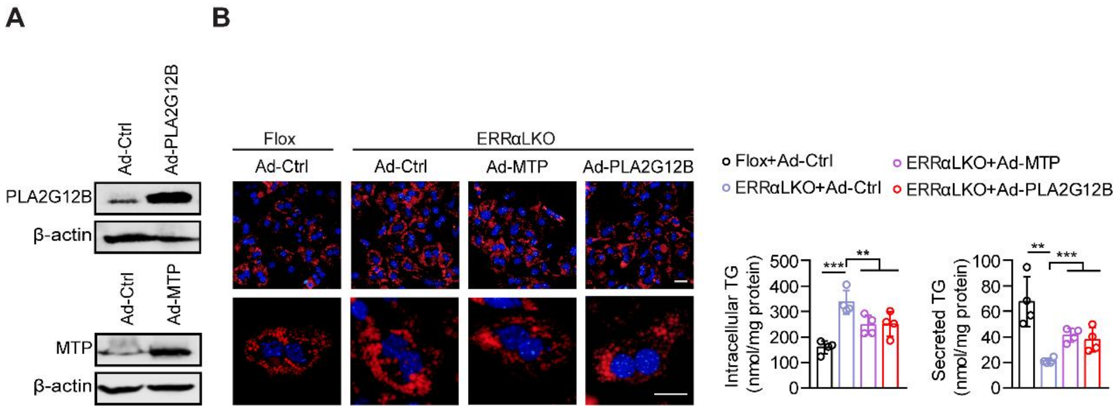

Figure S4. Restoration of MTP or PLA2G12B expression rescues VLDL-TG secretion in ERRαLKO hepatocytes. (A) Western blot showing the levels of MTP and PLA2G12B proteins in hepatocytes infected with adenovirus expressing MTP (Ad-MTP) or PLA2G12B (Ad-PLA2G12B). (B) Representative fields of male Flox and ERRαLKO hepatocytes infected with Ad-MTP, Ad-PLA2G12B or Ad-Ctrl. Lipid droplets (LDs) were labeled with Nile Red (red). Scale bar, 20 μm. Intracellular TG contents and secreted TG from hepatocytes infected with Ad-MTP, Ad-PLA2G12B or Ad-Ctrl as indicated (n = 4). Data presented as means ± SD. $^{**}P < 0.01$, $^{***}P < 0.001$, two-tailed Student's t test.

Table S1. The primers used to genotype the liver-specific ERRα-deficient mice.

Primer name	Sequence 5'---3'
Esrra-P1	CTGGAGAAAACTGAGGAGAGGA
Esrra-P2	ATGCTCCTAGTTCCATTCCAAA
Cre-P1	TGCAAACATCACATGCACAC
Cre-P2	TTGGCCCCTTACCATAACTG
Cre-P3	GAAGCAGAAGCTTAGGAAGATGG

Table S2. Mouse primers for quantitative real-time PCR detection.

Primer name		Sequence 5'---3'
Apob	F	GCCCATTGTGGACAAGTTGATC
	R	CCAGGACTTGGAGGTCTTGGA
Mttp	F	CAAGCTCACGTACTCCACTGAAG
	R	TCATCATCACCATCAGGATTCCT
PLa2g12b	F	CCAGCAATGACCAAGTGTTG
	R	GAGAATCACAGGCTGCTTCC
Acadm	F	AGGGTTTAGTTTTGAGTTGACGG
	R	CCCCGCTTTTGTCATATTCCG
Acaca	F	GATGAACCATCTCCGTTGGC
	R	GACCCAATTATGAATCGGGAGTG
Acacb	F	CGCTCACCAACAGTAAGGTGG
	R	GCTTGGCAGGGAGTTCCTC
Srebf1	F	GCAGCCACCATCTAGCCTG
	R	CAGCAGTGAGTCTGCCTTGAT
Fasn	F	GGCATCATTGGGCACTCCTT
	R	GCTGCAAGCACAGCCTCTCT
Scd1	F	TTCTTGCGATACACTCTGGTGC
	R	CGGGATTGAATGTTCTTGTCGT
Actin	F	GGCTGTATTCCCCTCCATCG
	R	CCAGTTGGTAACAATGCCATGT
Ppargc1α	F	TATGGAGTGACATAGAGTGTGCT
	R	CCACTTCAATCCACCCAGAAAG
Dgat2	F	GCGCTACTTCCGAGACTACTT
	R	GGGCCTTATGCCAGGAAACT
Pdi	F	AAAGCTGCCGCAAAACTGAAG
	R	GCAGTGTCAGACAGGGTTGTA
Cideb	F	CAATGGCCTGCTAAGGTCAGT
	R	TTCCAGACCCTACGGCTTAAC

Table S3. Human primers for quantitative real-time PCR detection

Primer name		Sequence 5'---3'
ESRRα	F	AGGGTTCCTCGGAGACAGAG
	R	TCACAGGATGCCACACCATAG
ApoB	F	CGGGCTCGTTACCACATGAAG
	R	GCGTAGAGACCCATCACATGATA
MTTP	F	AGCTCACGTACTCCACTGAAG
	R	CCTCCATAGTAAGGCCACATCC
PLA2G12B	F	TGGGCATTCCAGCAATGACAA
	R	GATCGAGTGGAGACACCATCG
ACTIN	F	CATGTACGTTGCTATCCAGGC
	R	CTCCTTAATGTCACGCACGAT
DGAT2	F	GCTGACCACCAGGAACTATATC
	R	CATTGCCACTCCCATTCTTTG
PDI	F	GGTGCTGCGGAAAAGCAAC
	R	ACCTGATCTCGGAACCTTCTG
CIDEB	F	CAGTGACTTACTCAGGTCAGTATC
	R	TTAGCACTCCATTCAGCAGTAG

Table S4. Primers for promoters

Primer name		Sequence 5'---3'
Apob WT	F	GGGGTACCTCTCAGCATCTTCTCTGCATCC
	R	CCGCTCGAGTGCTTGCTCTACAGGGTCTTC
Apob mA	F	GATGCTAACAATATGCCATGGGCTTCCTCTG
	R	CAGAGGAAGCCCATGGCATATTGTTAGCATC
Apob mB	F	CCGTGCCATTTGGAACTTTGGCAATTCTCC
	R	GGAGAATTGCCAAAGTTCCAAATGGCACGG
Apob ΔAB	F	GGGGTACCTGGCAATTCTCCTTAAGCCAGC
	R	CCGCTCGAGTGCTTGCTCTACAGGGTCTTC
Mttp WT	F	CCGGGTACCTATATGGCCACCCGGTATCCA
	R	CCGCTCGAGTAACGGAAGCAGAGTAGGAGGA
Mttp mA	F	GGAGGTTAAGGTAATTTTCGCCGTTCAATTTTGC
	R	GCAAAATTGAACGGCGAAAATTACCTTAACCTCC
Mttp ΔAB	F	CCGGGTACCGTAGCTCTATTGCTTGCTATC
	R	CCGCTCGAGTAACGGAAGCAGAGTAGGAGGA
Mttp mC	F	GGAGTTTGGAATCTGATCGTTCCCCTATAG
	R	CTATAGGGGAACGATCAGATTCCAAACTCC
Mttp ΔABC	F	CCGGGTACCAGATAAACACTGTTGTCGCCGG
	R	CCGCTCGAGTAACGGAAGCAGAGTAGGAGGA
PLa2g12b WT	F	CCGGGTACCGGAGTCCTATGGTTCTGTTCTG
	R	CCGCTCGAGTTTGCACGCACAAGTAGACC
PLa2g12b mA	F	CTGATATGATATCATCAGTTATGGCCCTGGCC
	R	GGCCAGGGCCATAACTGATGATATCATATCAG
PLa2g12b mB	F	CCTCCCACGGTTTAAAATTTATAATCTCTAATCGCTCG
	R	CGAGCGATTAGAGATTATAAATTTTAAACCGTGGGAGG
PLa2g12b ΔAB	F	CCGGGTACCGGATCGGCTCCCAGCCTCCA
	R	CCGCTCGAGTTTGCACGCACAAGTAGACC

Table S5. Primers for ChIP real-time PCR detection.

Primer name		Sequence 5’---3’
Esrra	F	CGAACCCAAGCTCAACCTCATCTG
	R	AGAGGTGAGACACGAGCGGACAAA
Apob site A	F	GAAATGACATCCCCCAATGATGC
	R	TTTGGAAATGGTTCTGCTTCCCC
Apob site B	F	GCAGTGAGACCACCATCAGATCC
	R	AGGCAGGTACTTGATCCACTGCA
Mttp site A	F	GTATATGGCCACCCGGTATCCACA
	R	GAAAGGTGAAAATTACCTTAACC
Mttp site B	F	GGAAAACACACTTCAAAGGACA
	R	GGAAGTGAGGGTTATTGGGATAT
Mttp site C	F	TGGCCTCACACTCAGAAATCCA
	R	GGGGAAAGGTCAGATTCCAAAC
PLa2g12b site A	F	GTGCTCAAGTGTGCTGGAGA
	R	GTTCCCCAGAGGTGGACTTT
PLa2g12b site B	F	CCCGTTTTCCTTCCTTTTTG
	R	CTTCCTCCCTCCACACATTC
